# Erythematous scaling lesions of the face, dorsal fingers, elbows, and knees together with symmetrical muscle weakness in a child

**DOI:** 10.1002/ccr3.2219

**Published:** 2019-05-29

**Authors:** Aleksander Markovic, Volker Schuster, Jan‐Christoph Simon, Manfred Kunz

**Affiliations:** ^1^ Department of Dermatology, Venereology and Allergology University of Leipzig Leipzig Germany; ^2^ Hospital for Children and Adolescents University of Leipzig Leipzig Germany

**Keywords:** autoimmune connective tissue diseases, immunological disorders, juvenile dermatomyositis, psoriasis

## Abstract

Juvenile dermatomyositis shows characteristic skin lesions. However, this does not rule out co‐occurring other autoimmune diseases, which may be more prominent regarding skin manifestations. Co‐occurring other skin autoimmune diseases should not be regarded as a preclusion for dermatomyositis. Here, we present an impressing case of juvenile dermatomyositis with co‐occurring psoriasis.

## 1 CASE REPORT

**Figure 1:**
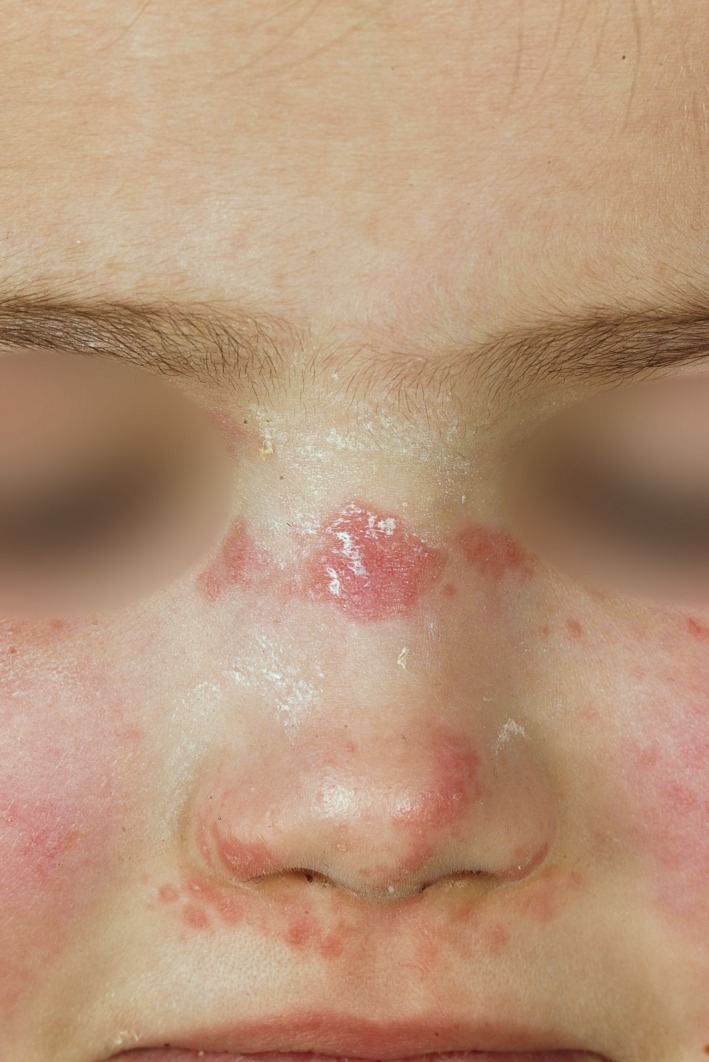
Erythematous plaques with scaling of the skin on the nose and cheeks (Photo M. Karsten/UKL)

**Figure 2:**
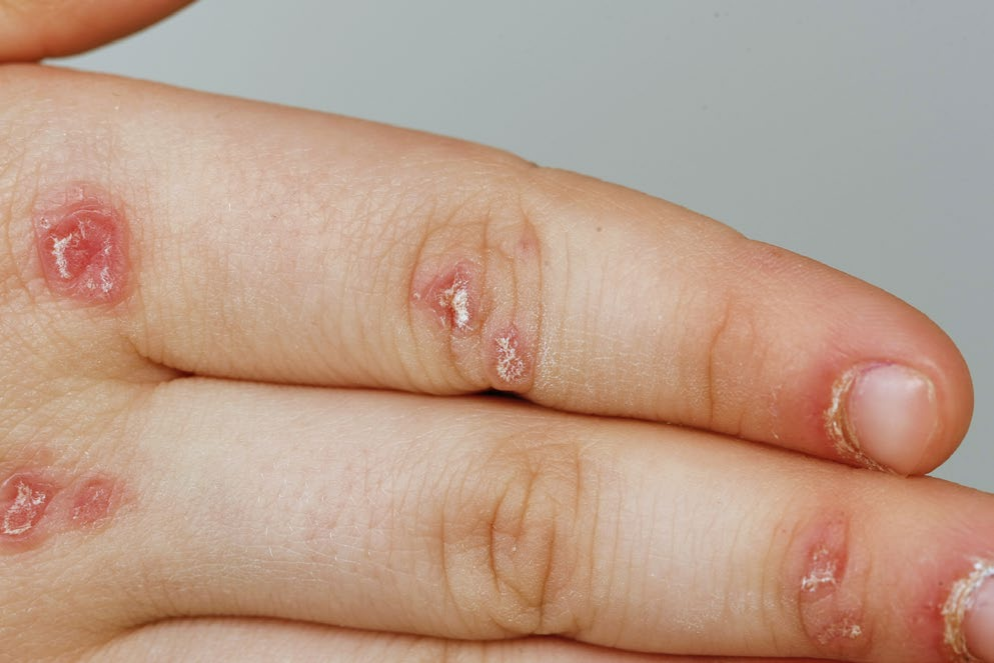
Right dorsal hand with scaling plaques and periungual papules of the fingers (Photo M. Karsten/UKL)

We present a 5‐year‐old girl with a recent onset of erythematous and scaling lesions of the face, dorsal fingers, elbows and knees, cuticular hypertrophy, and periungual capillary dilatation (Figures 1, 2). The patient also described a symmetrical pain and slowly increasing muscle weakness of her lower and upper extremities. After several days, muscle weakness worsened so that she was no longer able to walk upright, but instead started to crawl. Before onset of the disease, the patient showed a normal development. The mother reported that during pregnancy, she developed premature contractions in the 33rd week, and during that period, she was treated for gonorrhea with fluoroquinolones. There was no family history for any other diseases.

**Figure 3:**
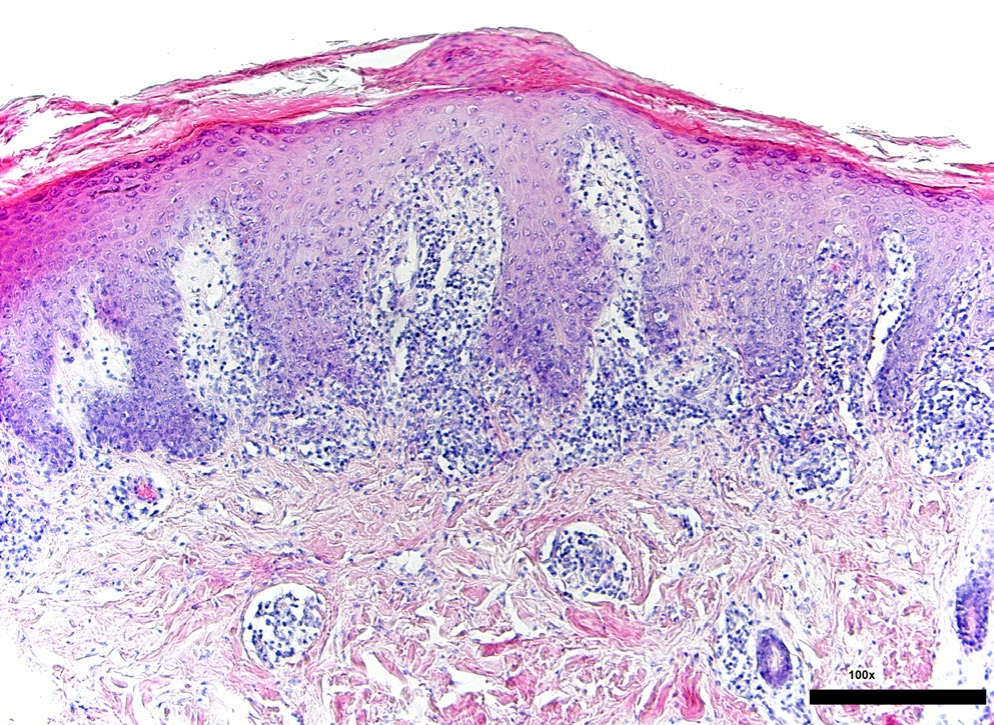
Epidermal acanthosis with regular elongation of the epidermal rete ridges. The suprapapillary epidermis is thinned with reduced or absent stratum granulosum and covered by parakeratosis. The upper dermal compartment shows a moderately dense inflammatory infiltrate of perivascular lymphocytes (Photo M. Ziemer/UKL)

A 4‐mm punch biopsy specimen of the skin of the left elbow was taken. The histopathological findings were typical for psoriasis (Figure 3).

Based on the physical complaints, neurological status, muscle ultrasound of the thighs which showed an echo‐rich activity of the muscles compatible with an inflammatory infiltration, laboratory testing (elevated creatine kinase levels [7.01 μkat/L, normal range <2/5 µkat/l]), lactate dehydrogenase levels (8.08 μkat/L, normal range <5.2 µkat/L), and a positive ANA titer of 1:2560, the diagnosis of dermatomyositis was made according to the European League Against Rheumatism (EULAR)/American College of Rheumatology (ACR) classification criteria for adult and juvenile idiopathic inflammatory myopathies.^1^

## 2 DISCUSSION

Juvenile dermatomyositis (JDM) is the most common myopathy in childhood.^2^ It is a vasculopathy which is associated with proximal muscle weakness and characteristic skin manifestations such as microvascular dilation, Gottron's papules over extensor joint surfaces, and a heliotrope rash around the eyes.^2^, ^3^ The etiology of the disease remains unknown. Juvenile dermatomyositis may present as an amyopathic as well as a myopathic variant.^3^ Characteristic features of JDM according to the latter survey are proximal muscle weakness (100%), arthralgia (55%), Gottron's papules (93%), heliotrope rash (87%), photosensitivity (55%), fatigue (82%), weight loss (40%), and abdominal pain (36%).^3^ In clinically amyopathic JDM (CAJDM) myalgias, arthritis, contractures, calcinosis, dysphagia, abdominal pain, fatigue, and blood and serum abnormalities are less frequent. However, CAJDM and classic juvenile dermatomyositis (JDM) share a high percentage of positive ANA titers (70%‐80%).^3^

Psoriasis is not uncommon in childhood and can be associated with a positive family history.^4^, ^5^ It is characterized by erythematous papules and plaques with characteristic scaling. Both diseases, dermatomyositis and psoriasis, appear to share different signaling pathways of TNF‐α and IFN‐α/β signaling.^5^ However, the role of IL‐23‐IL17‐axis signaling, which is of central importance in psoriasis, is still controversially discussed in dermatomyositis. Interestingly, there are a small number of reports presenting both diseases in one patient at young age, similar to our case.^5^ All three patients in the report by Nikki and co‐authors were under the age of 18 and showed proximal muscle weakness and were treated with a combination therapy of methotrexate and methylprednisolone. In addition, one report demonstrates a patient at the age of 20 who had amyopathic juvenile dermatomyositis with psoriasis.^6^ Current treatment options recommend the use of a steroid‐pulse therapy with 30 mg/kg with a 1‐g maximum dosage on several occasions in conjunction with oral administration of prednisone 0.5 mg/kg/d alone, or in combination with other immunosuppressants such as methotrexate.^5^ If prednisone is given, a cushingoid body habitus and hypertrichosis may develop as side effects like in our patient (not shown). We added 15 mg/m^2^/wk methotrexate to reduce steroid dosage, as has been described elsewhere.^5^

Due to a relatively safe profile, treatment options with TNF‐α antagonists such as infliximab or etanercept may be used in children with treatment‐resistant dermatomyositis.^7^

## CONFLICT OF INTEREST

None declared.

## AUTHOR CONTRIBUTIONS

Aleksander Markovic: had full access to all the data in the study and takes responsibility for the integrity of the data and the accuracy of the data analysis. Aleksander Markovic, Volker Schuster, and Manfred Kunz: involved in study concept and design. Aleksander Markovic, Volker Schuster, Jan C. Simon, and Manfred Kunz: acquired, analyzed, and interpreted the data. Aleksander Markovic, Jan C. Simon, and Manfred Kunz: drafted the manuscript. Aleksander Markovic, Jan C. Simon, and Manfred Kunz: involved in critical revision of the manuscript for important intellectual content. Aleksander Markovic, Jan C. Simon, and Manfred Kunz: served as administrative, technical, or material support. Aleksander Markovic and Manfred Kunz: supervised the study.
